# Comparative Analysis of Deep Learning Methods on CT Images for Lung Cancer Specification

**DOI:** 10.3390/cancers16193321

**Published:** 2024-09-28

**Authors:** Muruvvet Kalkan, Mehmet S. Guzel, Fatih Ekinci, Ebru Akcapinar Sezer, Tunc Asuroglu

**Affiliations:** 1 Department of Computer Engineering, Ankara University, 06830 Ankara, Turkey; kalkanm@ankara.edu.tr (M.K.); mguzel@ankara.edu.tr (M.S.G.); 2 Department of Institute of Nuclear Sciences, Ankara University, 06100 Ankara, Turkey; fatihekinci@ankara.edu.tr; 3 Department of Computer Engineering, Hacettepe University, 06800 Ankara, Turkey; ebru@hacettepe.edu.tr; 4 Faculty of Medicine and Health Technology, Tampere University, 33720 Tampere, Finland; 5 VTT Technical Research Centre of Finland, 33101 Tampere, Finland

**Keywords:** lung cancer, classification, CNN, deep learning, segmentation, CT images

## Abstract

**Simple Summary:**

Lung cancer is the most common form of cancer globally, making early detection essential for improving patient outcomes. In this study, deep learning methods were used to analyze CT lung images to detect cancer at an early stage. Several pre-trained models were employed, with InceptionResNetV2 achieving the highest detection accuracy of 98.5%. When cancer was detected, the extent of the tumor area was determined through segmentation models. The most accurate results were obtained using the InceptionUNet model, which achieved a Jaccard index of 95.3% in identifying the tumor area. These findings demonstrate the potential of deep learning for enhancing both the detection and localization of lung cancer, which may contribute to more effective treatment strategies.

**Abstract:**

Background: Lung cancer is the leading cause of cancer-related deaths worldwide, ranking first in men and second in women. Due to its aggressive nature, early detection and accurate localization of tumors are crucial for improving patient outcomes. This study aims to apply advanced deep learning techniques to identify lung cancer in its early stages using CT scan images. Methods: Pre-trained convolutional neural networks (CNNs), including MobileNetV2, ResNet152V2, InceptionResNetV2, Xception, VGG-19, and InceptionV3, were used for lung cancer detection. Once the disease was identified, the tumor’s region was segmented using models such as UNet, SegNet, and InceptionUNet. Results: The InceptionResNetV2 model achieved the highest detection accuracy of 98.5%, while UNet produced the best segmentation results, with a Jaccard index of 95.3%. Conclusions: The study demonstrates the effectiveness of deep learning models, particularly InceptionResNetV2 and UNet, in both detecting and segmenting lung cancer, showing significant potential for aiding early diagnosis and treatment. Future work could focus on refining these models and exploring their application in other medical domains.

## 1. Introduction

Lung cancer is a condition marked by the abnormal and uncontrolled division of lung cells. This type of cancer can lead to significant physical harm and may be life-threatening. Due to its widespread occurrence globally, lung cancer has become a significant health issue threatening human well-being. According to the research conducted in 2022, there were nearly 2.5 million new patients diagnosed with lung cancer, and more than 1.8 million people lost their lives to the disease worldwide within the same year [1].

Given that lung cancer spreads rapidly and can quickly affect other organs, early detection is crucial. Treating advanced lung cancer is both challenging and complex, making early diagnosis and identification of the tumor area critical for effective treatment. For instance, a recent case study highlighted that soft tissue metastases from lung cancer can mimic other conditions, complicating diagnosis [2]. Deep learning algorithms have the potential to detect serious diseases like lung cancer in their early stages, thereby contributing to saving human lives. By training neural models on a dataset of lung CT scans, cancerous cells can be accurately identified with high precision and sensitivity. Additionally, these models can quickly scan and analyze radiology images, often capturing fine details that may be overlooked by the human eye. Given the aggressive nature of lung cancer and the challenges associated with its late-stage treatment, this study underscores the critical need for early detection to improve patient outcomes and survival rates. The novelty of this research lies in the development and application of a two-stage deep learning approach that not only classifies lung CT images with high accuracy but also precisely segments the tumor region, providing a comprehensive tool for early and accurate diagnosis.

**Figure 1:**
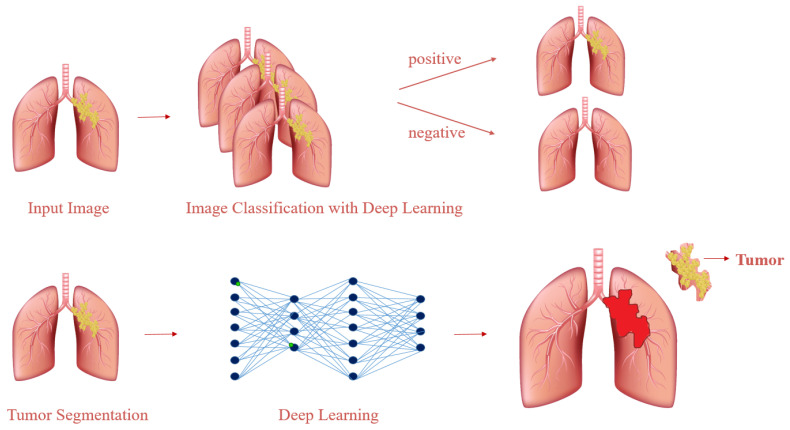
Deep-learning-based lung cancer diagnosis.

The study involved a two-stage detection process primarily using medical images. In the first stage, the aim was to detect the disease by classifying it using deep learning algorithms. In the second stage, segmentation was performed on the images where the disease was detected in order to determine the region of the tumor, as illustrated in Figure 1. From the experiment, satisfying results were produced in which the approach proposed can classify CT images as healthy/sick, then predict tumor region accurately, as given in the following sections in detail.

From the experiment, satisfying results were produced in which the approach proposed can classify CT images as healthy/sick then predict tumor region accurately, as given in the following sections in detail. The Literature Review section outlines the prior research related to the applied methodology and comparable studies from the past. The Methodology section explores the details of the dataset and the methods used, including the models and experimental steps. The Experimental Results section outlines the findings of the experiment and explains the criteria used for their evaluation, specifically the metrics. The Discussion section then interprets these results and draws certain conclusions. Finally, the Conclusion provides a summary of the overall study, including the results and key takeaways.

## 2. Literature Review

In the recent decades, the use of AI technologies like deep learning, image classification, and segmentation has notable increased across multiple domains. In healthcare, these technologies have notably accelerated the diagnostic process and greatly minimized errors in calculating tumor areas by analyzing multiple images.

### 2.1. Model Foundations

For this experiment, certain pre-trained models were chosen to classify lung images and differentiate between diseased and healthy states: MobileNetV2, ResNet152V2, Xception, InceptionResNetV2, VGG-19, and InceptionV3.

MobileNets, developed by Google researchers with a focus on mobile applications, are engineered to be efficient, compact, and versatile [3,4]. The architecture reduces computational costs by employing depthwise separable convolution layers [5,6,7].

ResNets, originally developed by Microsoft researchers, are designed to create deeper neural network architectures and address the challenges posed by deep layers through “residual mapping” [8,9]. ResNet152V2, a model that utilizes residual connections, enhances deep convolutional neural networks (CNNs). Having a depth of 152 layers, ResNet152V2 is highly effective in image recognition and classification tasks [10].

Xception, created by F. Chollet at Google, extends the Inception model framework to its limits, resulting in a model that surpasses the original Inception models, hence the name “Extreme Inception” [11]. By using depthwise separable convolutions instead of standard ones, Xception becomes more efficient and powerful. It enhances CNN performance by more effectively distinguishing spatial and cross-channel correlations within feature maps, supporting activities like image categorization and object recognition [12].

InceptionResNetV2, developed by Christian Szegedy and his team at Google, combines residual connections with the Inception framework, enhancing feature extraction and overcoming training difficulties associated with very deep networks. This makes InceptionResNetV2 a robust and effective architecture for various image recognition tasks [13].

In 2014, VGG—abbreviated form of “Visual Geometry Group”—networks are of a specific advanced type created at Oxford University. VGG-19 has a reputation of a robust performance in tasks of computer vision, despite its high depth of 19 layers. VGG-16 or VGG-19 are preferred popularly in tasks requiring image classification, feature extraction, and object recognition, leveraging a large number of small 3 × 3 convolutional filters to learn deep and complex features, leading to high accuracy and performance [14,15].

Inception architectures, developed by Google engineers and researchers, feature inception blocks—specialized components that use parallel convolutional layers, culminating in a concatenation layer that merges outputs into a single result. Inception models tackle the issues posed by extremely deep networks by assessing loss at mid-level layers and combining these with the overall loss. [16].

Ronneberger and his colleagues proposed the UNet model in 2015, which UNet is an image segmentation model prepared for biomedical visuals. It delivers detailed and accurate segmentation, demonstrating high performance even with limited data, particularly in tasks like cell and organ segmentation [17,18].

The SegNet model, introduced by Vijay Badrinarayanan, Alex Kendall, and Roberto Cipolla from the University of Cambridge, is also designed for image segmentation. It features a encoder–decoder structure within a deep CNN that efficiently classifies single pixels of images. SegNet’s unique upsampling method uses pooling indices from the encoder to enhance segmentation accuracy, making it particularly effective for precise segmentation tasks such as medical imaging and autonomous driving [19,20].

These models were chosen for the two-stage experiment, taking into account their conceptual and structural differences. This approach allows for a comparative analysis at each stage, enabling an interpretation of how the models’ underlying concepts and structures influence their performance on the task.

### 2.2. Related Works

In their work, Punn et al. employed the InceptionUNet architecture to automate nuclear detection in microscope cell images. This architecture integrates switch normalization layers, convolution layers, and pooling layers (max and Hartley spectral pooling) with 1 × 1, 3 × 3, and 5 × 5 convolutions within the UNet scheme. They also used activation maximization and filter map visualization techniques to analyze the model’s performance. InceptionUNet was shown to significantly outperform the original UNet and UNet++ models on the KDSB18 dataset [21,22,23,24].

Numerous academic studies have explored this subject. Tekade and Rajeswari developed a system for detecting lung nodules from tomography images with high accuracy and predicting malignancy levels using a combination of UNet and a 3D multipath VGG-like network. This approach has proven effective in predicting lung cancer risk, achieving a 95.66% accuracy rate [25].

In a different study, Sharma and his team introduced a two-step approach to detect early cancer in lungs, utilizing data-driven segmentation and deep CNNs combined with the Otsu technique. Their method outperformed the existing algorithms, achieving 84.13% accuracy on CT images [26].

Likewise, Lakshmanaprabu et al. discerned malign and benign nodules in lungs employing an “Optimal Deep Neural Network (ODNN)” with the use of “Linear Discriminant Analysis (LDA)”. Achieving 94.56% accuracy on lung tomography images, their method provides a quick, straightforward, and cost-effective solution by minimizing manual labeling errors [27].

Jafar and his colleagues designed and used a customized “Weighted Optimized Neural Network (WONN)” to diagnose images with lung cancer from large datasets. The researchers designed a combination of their model with “Maximum Likelihood Boosting (MLB)”, named “WONNN-MLB”. The approach enhances accuracy and lowers false positive rates. This method facilitates faster and more accurate diagnoses by integrating feature selection with enhanced neural network classification [28].

To tackle the challenges of detecting various lung tumors, Lu and his co-researchers proposed a novel “Computer-Aided Diagnosis (CAD)” system. They used 294 lung CT radiology images from “Lung Image Database Consortium (LIDC)” and divided them into groups of training and testing, with sizes of 196 and 98, respectively. A total of 631 nodules in lung images were identified by two radiologist medical doctors, with sensitivity measured at 87% during training and 85.2% during testing [29].

Manikandan and Bharathi created a “Fuzzy Auto-Seed Cluster Means Morphological Algorithm” to detect cancer by segmentation of lung nodules from CT scans. They determined initial cluster values by averaging pixel values in the rows and removed blood vessels through center change analysis. Using SVM, they classified real malignant nodules by calculating texture features in nodules. The study, conducted on cases with 56 of malignant nodules and 745 of benign nodules, achieved 100% sensitivity, 93% specificity, and 94% accuracy. The false positive rate (FPR) for each patient was found to be 0.38 [30].

Wang and colleagues came forward with a novel model called “Center Focused Convolutional Neural Networks (CF-CNN)” to precisely perform segmentation to find lung nodules on CT scans, taking a different approach to CNN architectures. CF-CNN captures features that are sensitive to nodules from both 2D and 3D scans and assesses neighboring voxel effects through a central pooling layer. Evaluated by processing the dataset of LIDC with 893 examples of nodules and a separate dataset from “Guangdong General Hospital (GDGH)” comprising 74 nodules, the model achieved a Dice–Sørensen coefficient score of 82.15% and 80.02%, respectively. This study emphasized the current potential of applying deep learning architecture in lung nodule segmentation and aimed to advance this approach in the future [31].

Teramoto and Fujita, aiming for swift lung tumor detection, proposed a novel method using a cylindrical nodule boosting filter. This method seeks to improve time efficiency on nodule detection by minimizing computational time. The proposed method follows the given steps. First segment lung regions, then preprocess, next augment nodules, follow with a further segmentation, and finally, reduce false positives. The nodule candidates are identified using a cylindrical filter, and false positives are reduced with seven characteristic parameters using support vector machines. Experiments on the LIDC dataset reveal that the method detects 80% of nodules with minimal false positives and shows at least 4 and at most 36 times better time efficiency than the current methods. The results suggest that this method could be beneficial to detect nodules on chest CT scans in clinical settings [32].

While the aforementioned studies offer valuable insights into lung cancer detection using deep learning, there remains a need for a combined approach that integrates both classification and segmentation, which is important for early treatment planning.

## 3. Methodology

CNNs have emerged as the preferred method for medical image classification tasks in recent times. In the experiment, pre-trained CNN architectures were utilized to classify CT images as either patient or healthy. While numerous studies exist in this area, this research offers a comprehensive approach that not only includes diagnosing the disease from images but also segments the tumor region if the disease is detected. Moreover, the results obtained were highly satisfactory.

**Figure 2:**
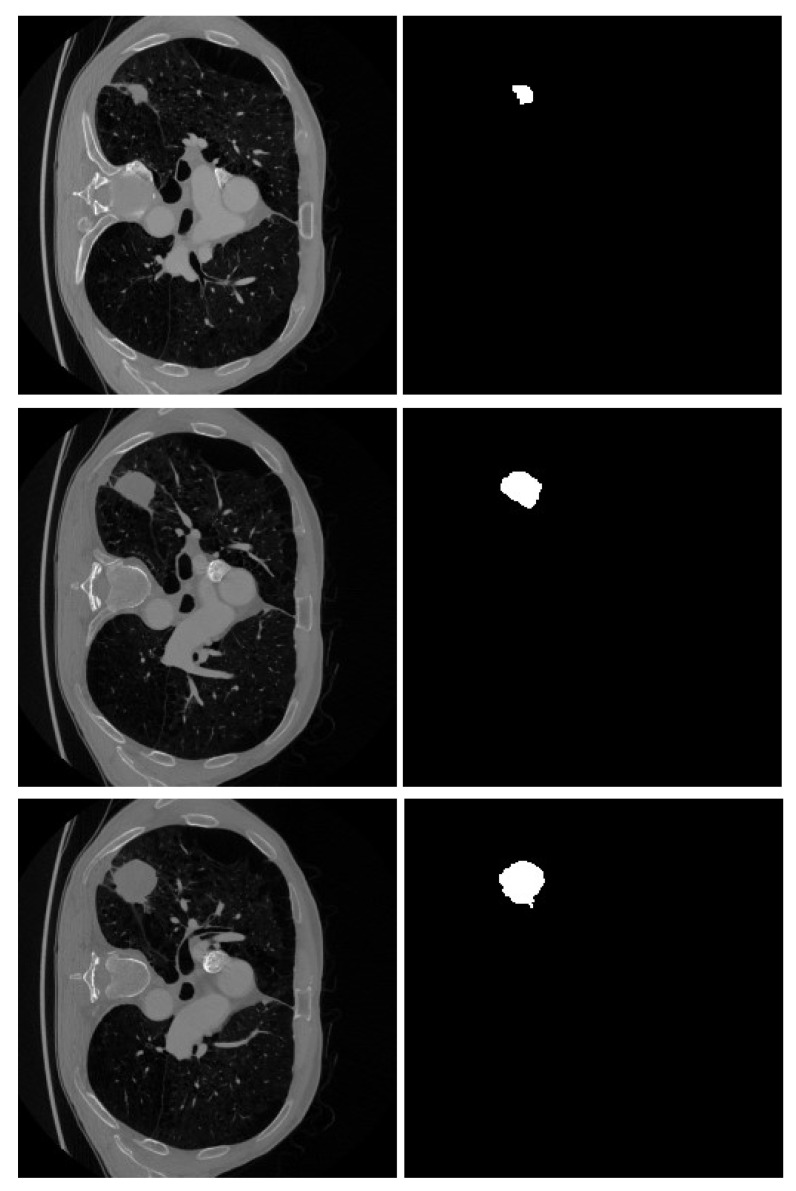
Some samples of lung CT images and their region of tumor as a black-white mask.

The experimental studies were conducted using data from a public Kaggle dataset [33]. A total of 3296 images were selected from this dataset, which contains over 14,000 images along with their corresponding masks. The initial step involved dividing the dataset into two categories: patient/healthy (positive/negative). Each image is paired with a corresponding mask image. These mask images are black-and-white and highlight the tumor region, if present, on the original image, as illustrated in the Figure 2.

The research was conducted using the Google Colab platform, which was chosen for its ability to fulfill the experiment’s technical requirements. Google Colab is cloud-based, does not require installation, and is freely accessible, making it ideal for collaborative work. It is commonly utilized for implementing machine learning and deep learning projects, as it supports Python and TensorFlow, a widely used open-source library that provides various CNN architectures and features.

The following procedures were followed to prepare the classification model for training.

1. **Scaling of Images:** First, the images are imported and resized to 128 × 128 pixels.
2. **Dataset Splitting:** The dataset, which consists of 3296 images, is split into three: 70% for training, 20% for validation, and lastly, 10% for testing.
3. **Data Augmentation:** The dataset size is artificially expanded through the augmentation process, increasing the original 2543 images to ten times their initial quantity.
4. **Pre-processing:** Each image consists of 1D matrices containing RGB values, which are then scaled to the range [0, 1] or [−1, 1]. The necessary pre-processing layer is defined for the pre-trained models to ensure the values are within the appropriate range.
5. **Models:** Pre-trained models loaded from the TensorFlow library are used as base models. These models consist of multiple layers.
6. **Pooling Layer:** A pooling layer is defined (Global Average).
7. **Prediction Layer:** The prediction layer is set up with the softmax activation function to produce the outputs of the model, as softmax offers the most accurate probability for each class membership.
8. **Model Compilation:** The primary model is constructed by combining these layers with the base model. Each of the seven pre-trained models serves as the foundation for its respective experiment, with identical procedures applied to each one. The structural details of the layers in the main models are provided in Table 1. After compiling, categorical cross-entropy loss is used, along with evaluation metrics such as accuracy, precision, recall, F1-Score, and ROC AUC.
9. **Training:** The main model is trained for 20 epochs, with the loss and metrics recorded at each epoch throughout the training process.
10. **Model Evaluation:** After training, the success of the main model, including base pre-trained model, is evaluated. The evaluation is performed by examining and comparing metric results from predicting the images of the test dataset. The assessment includes accuracy, cross-entropy loss, precision, recall, F1-Score, and ROC AUC. These outcomes are subsequently utilized to compare the overall performance of the primary models.

The following steps were applied to prepare the segmentation model in its trained form.

**Figure 3:**
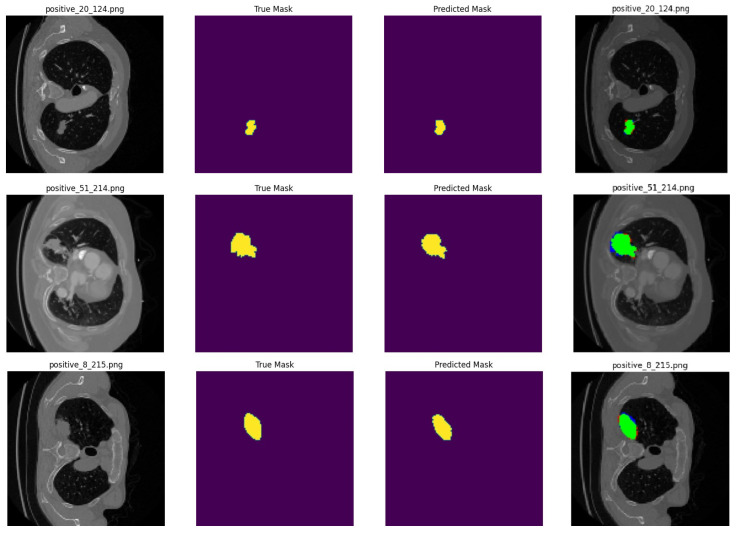
The sample prediction results are presented in the following order: first, the original image with its filename; second, the true mask of the tumor region; third, the predicted mask generated by the segmentation model; and finally, a combined image displaying all three. In the combined image, the masks are overlaid onto the original image, with green indicating correctly predicted tumor areas, blue showing missed actual tumor regions, and red highlighting healthy areas mistakenly classified as tumor.

1. **Image Preparation:** The images were rescaled to a size of 128 × 128 pixels.
2. **Data Augmentation and Splitting:** The dataset was split into parts of 70:20:10 percents for training, validation, and test, respectively.
3. **Model Preparation:** A model was constructed for the segmentation process, with one of the UNet, SegNet, or Inception UNet models selected and used. Encoder–decoder connections were established, as outlined in Table 2.
4. **Training:** The segmentation architecture is trained with 20 epochs, with the loss and metric results recorded at each epoch during the training process.
5. **Model Evaluation:** The final metric results are calculated using the test dataset, which was not included in the training phase.
6. **Visualization:** The input image, the actual mask, and the predicted mask from selected test samples are displayed, as given in Figure 3. These steps are repeated for each of the other segmentation models.

## 4. Experimental Results

The entire study was carried out in Google Colab’s cloud environment, utilizing the platform’s provided hardware. The experiment made use of the Google TPU V2, a specialized processing unit designed specifically for matrix calculations, which are fundamental to the operations of neural networks.

As previously discussed, the trained models produce predictions on randomly selected samples from the test data. This particular dataset was chosen because it contains information not seen by the models during training, ensuring unbiased behavior and providing more objective outcomes. The predictions are presented as 1D arrays or vectors since the softmax is used as the activation function in the final prediction layer across all primary models, enabling categorical classification. Each array’s length corresponds to the number of classes—eleven in this study. Every element within the array represents a probability value ranging from zero to one ([0, 1]), with the sum of all these floating numbers equal to one. Every index is associated with a particular class, and the value at that index indicates the likelihood that the input image is classified under that class. The model’s final prediction is determined by the class with the greatest probability.

Once the classification process identifies the test images, the input image associated with a patient is forwarded for segmentation. The encoder layers compress (encode) the input image into a lower-dimensional representation by passing it through multiple convolution layers, where features are extracted. In the bottleneck layer, the smallest feature map from the encoder undergoes deeper convolution, enhancing the model’s learning capability. The decoder then combines (decodes) these compressed data with the encoder layers to reconstruct the output image in its original dimensions. Finally, an output layer is utilized to estimate each pixel’s likelihood of belonging to a specific class. The outcome includes the original image, the true mask, and the estimated mask generated after processing.

For all classification models, both training and testing scores are generated and documented using six metrics for comparison: accuracy, cross-entropy loss, precision, recall, F1-score, and ROC AUC.

In the case of segmentation models, six metrics are computed and contrasted throughout the training process and for the test data: Jaccard index, accuracy, cross-entropy loss, precision, recall, and F1-score. The metrics are thoroughly examined in this section and further elaborated in the Discussion section below.

All metrics, except for cross-entropy loss, are calculated using the prediction system’s output. When predicting positive instances from a set containing both positive and negative values, each prediction is categorized as either true or false. The following values are derived based on these predictions:

*TP*: True Positives—the number of correctly predicted positive cases.
*FP*: False Positives—the number of incorrectly predicted positive cases, which are actually negative.
*TN*: True Negatives—the number of correctly predicted negative cases.
*FN*: False Negatives—the number of incorrectly predicted negative cases, which are actually positive.
  (1) $$Accuracy=\frac{TN+TP}{TP+TN+FN+FP}$$

Accuracy is calculated as the proportion of the model’s correct predictions (TP and FP) relative to the number of all the predictions. Simply put, it represents how frequently the model makes accurate predictions, as illustrated in Equation (1).

(2) $$Precision=\frac{TP}{FP+TP}$$

Precision quantifies the accuracy of the model’s positive predictions (TP) over the predicted positives (TP and FP). Specifically, it evaluates how well the model correctly identifies positive cases, as illustrated in Equation (2).

(3) $$Recall=\frac{TP}{TP+FN}$$

Recall represents the fraction of TP instances that the model accurately detects over the actual positives (TP and FN), as demonstrated in Equation (3).

(4) $$F_{1}=\frac{TP}{TP+\frac{1}{2}\left(FP+FN\right)}$$

When precision and recall are significantly imbalanced, and it is essential to achieve equilibrium between these metrics, the F1 score becomes crucial. The F1 score is particularly useful when reducing all false predictions. The formula for the metric F1-Score is provided in Equation (4).

(5) $$TPR=\frac{TP}{TP+FN}$$

(6) $$FPR=\frac{FP}{FP+FN}$$

**Figure 4:**
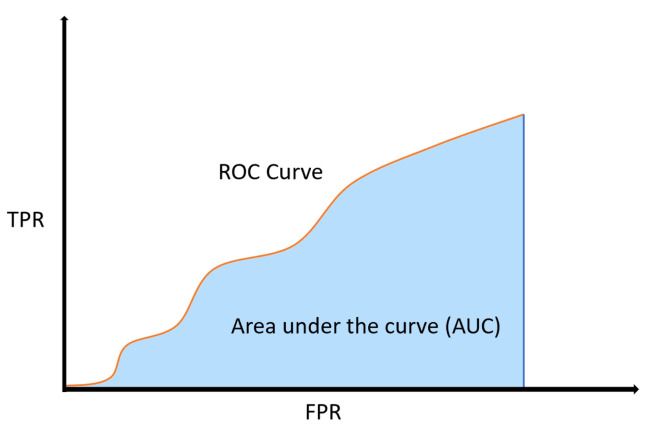
ROC curve and its AUC.

The ROC curve is a graph taking the true positive rate (TPR), Equation (5), as y-axis and the false positive rate (FPR), Equation (6), as x-axis. The area under the curve (AUC) represents the area beneath this curve. A higher AUC signifies a stronger overall ability of the model to differentiate positive and negative prediction sets from one another. The higher the value of ROC AUC, the better the model performs, as shown in Figure 4.

Loss is a numerical measurement of the distance between actual classes and the predicted results. The loss is calculated using this distance in a logarithmic way. In both classification and segmentation tasks, the categorical type of cross-entropy loss is preferred due to the multi-class nature of them. The loss function directs the model’s learning by striving to minimize errors. A lower loss value reflects better model performance.

In the segmentation phase, the Jaccard index measures the ratio of the intersection of two clusters to their union. Known as “Intersection over Union (IoU)” as well, this metric is widely used in segmentation tasks. The Jaccard index varies between 0 and 1, with 1 signifying complete overlap between the clusters and 0 indicating no overlap. A high Jaccard index means that the segments predicted by the model closely match the actual segments. This metric is particularly valuable for evaluating the accuracy of segmented areas in an image, as illustrated in Equation (7).

(7) $$Jaccard\;Index=\frac{\left|A\cap B\right|}{\left|A\cup B\right|}=\frac{TP}{TP+FP+FN}$$

Training occurs over several epochs, with evaluation metrics calculated at each step to generate learning curves. All the selected models are put to training on both the training and validation datasets, enabling the visualization of key metrics through these curves.

**Figure 5:**
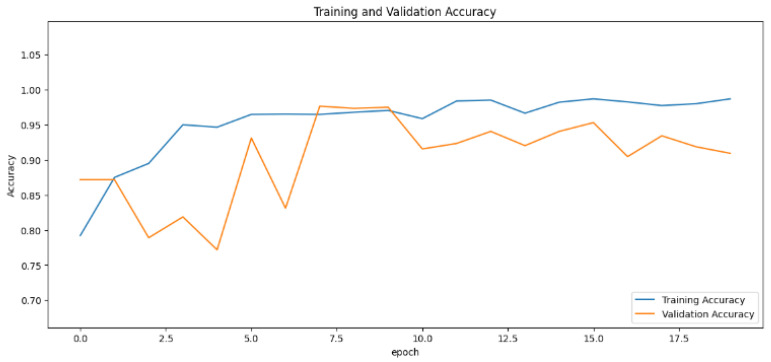
Accuracy rate of the MobileNetV2-based model by epochs, presented as learning curves.

**Figure 6:**
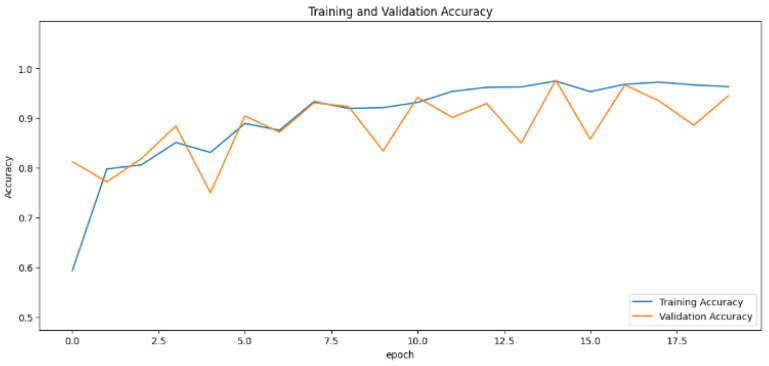
Accuracy rate of the VGG-19-based model by epochs, presented as learning curves.

**Figure 7:**
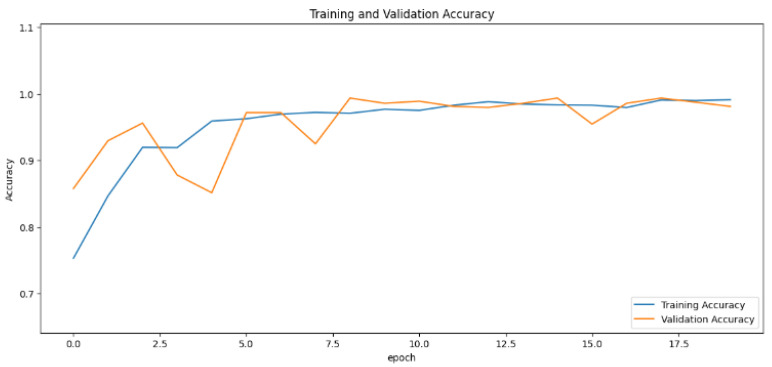
Accuracy rate of the ResNet152V2-based model by epochs, presented as learning curves.

**Figure 8:**
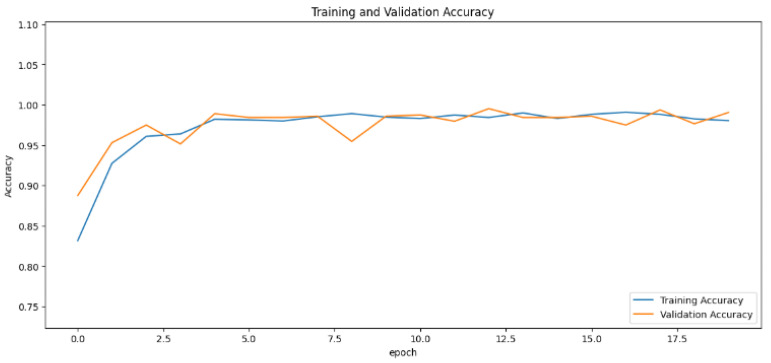
Accuracy rate of the Xception-based model by epochs, presented as learning curves.

**Figure 9:**
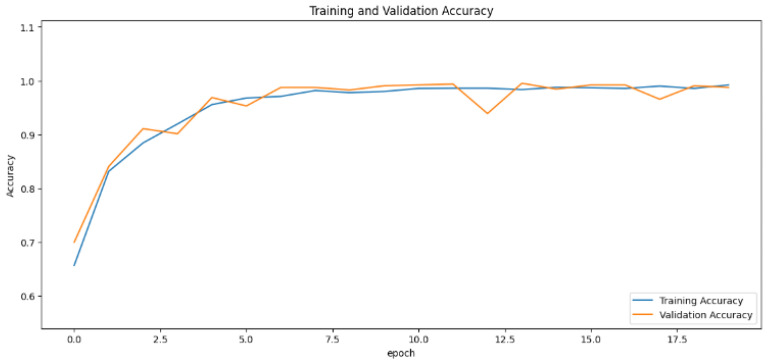
Accuracy rate of the InceptionResNetV2-based model by epochs, presented as learning curves.

**Figure 10:**
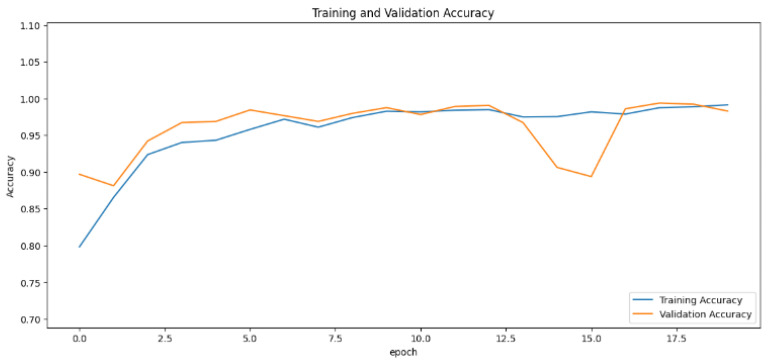
Accuracy rate of the InceptionV3-based model by epochs, presented as learning curves.

The accuracy values for all models and datasets during the classification phase vary depending on the specific model. Observations from the learning curves reveal distinct patterns. The learning curves of the models MobileNet V2 and VGG-19 models indicate that the learning and mastery process for these models is generally moderate, as shown in Figure 5 and Figure 6 with high–low oscillations in validation. The ResNet152V2 model exhibited a gradual increase in accuracy during the initial training periods, followed by stability, as depicted in Figure 7. For the Xception, InceptionResNetV2, and InceptionV3 models, a significant jump in accuracy was observed early in the training process, and these models maintained their accuracy rates with very small oscillations thereafter, as seen in Figure 8, Figure 9 and Figure 10.

**Figure 11:**
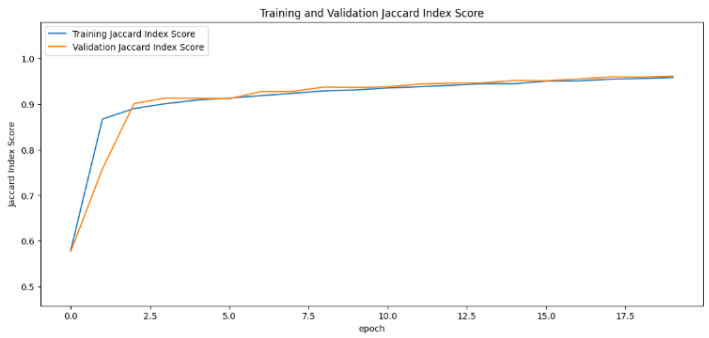
Jaccard index rate of the UNet-based model by epochs, presented as learning curves.

**Figure 12:**
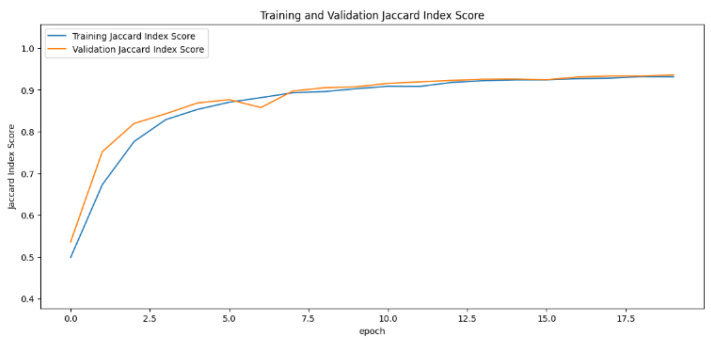
Jaccard index rate of the SegNet-based model by epochs, presented as learning curves.

**Figure 13:**
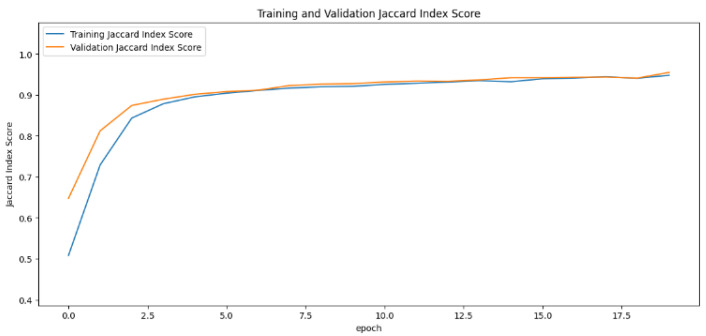
Jaccard index rate of the InceptionUNet-based model by epochs, presented as learning curve.

In the segmentation phase, the Jaccard index values generated by the training and validation datasets fluctuate over different time periods. This metric, essential for assessing the success of segmentation, was analyzed as the primary metric using learning curves. The learning curve for the UNet model displayed a sharp increase, indicating rapid improvement. In contrast, the SegNet model remained mostly stable, with minor fluctuations at certain points. The InceptionUNet model, on the other hand, showed a more gradual increase in its values with the smoothest transitions. For a detailed comparison, refer to Figure 11, Figure 12 and Figure 13.

**Figure 14:**
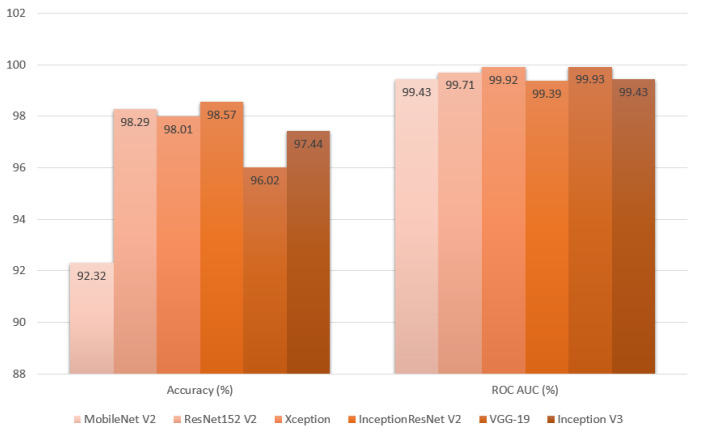
Classification metrics of accuracy and ROC AUC produced from final evaluation presented as a bar graph.

**Figure 15:**
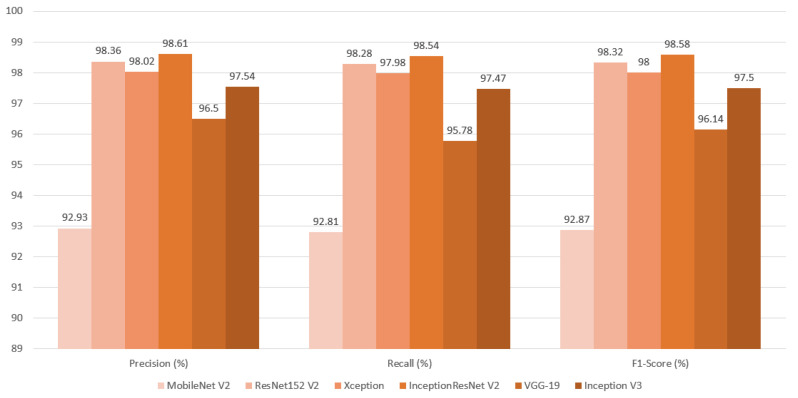
Classification metrics of F1-score, precision, and recall produced from final evaluation presented as a bar graph.

**Figure 16:**
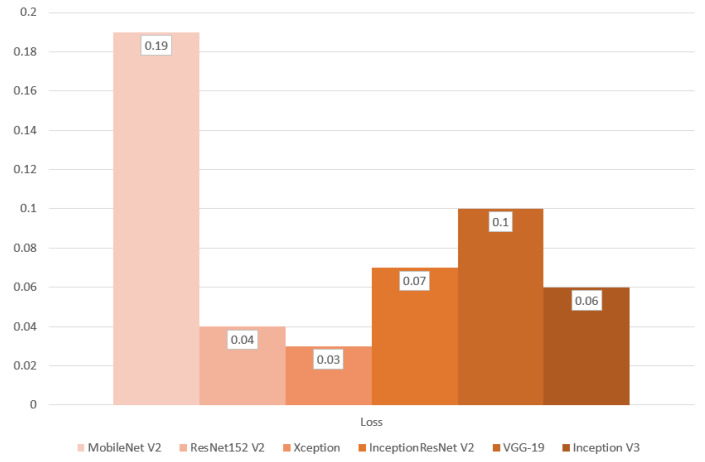
Classification metric of loss produced from final evaluation presented as a bar graph.

In the final evaluation section, the results from the test dataset were thoroughly analyzed, enabling an objective comparison of each metric based on the model used in the initial stage of image classification. The InceptionResNetV2 model achieved the highest accuracy at 98.57%, while the Xception model recorded the lowest loss value at 0.03. When assessing precision (98.61%), recall (98.54%), and F1 score (98.58%), the InceptionResNetV2 model again delivered the best results. Additionally, the VGG-19 model achieved the highest ROC AUC value at 99.93%. Overall, the InceptionResNetV2 model emerged as the top performer across all metrics, with the exception of the ROC AUC value. For a detailed comparison, refer to Table 3, along with Figure 14, Figure 15 and Figure 16.

**Figure 17:**
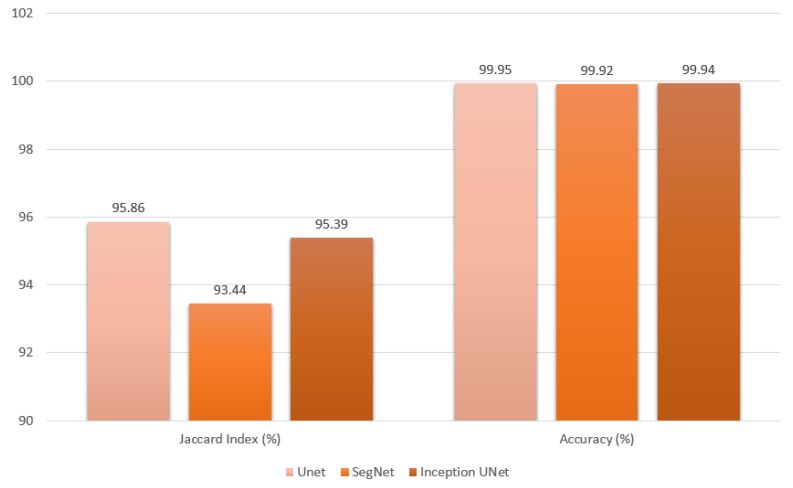
Segmentation metric of Jaccard index produced from final evaluation presented as a bar graph.

**Figure 18:**
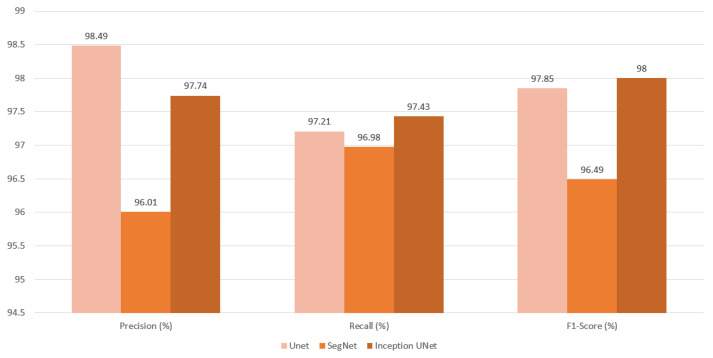
Segmentation metrics of F1-Score, precision, and recall produced from final evaluation presented as a bar graph.

The test dataset, generated from the classification process, proceeds to the segmentation stage, where the results are evaluated using various metrics. During this evaluation, each metric is objectively analyzed according to the selected model. The UNet model achieved the highest performance, with a Jaccard Index value of 95.86%. The InceptionUNetV2 model followed closely with 95.39%, while the SegNet model also exhibited strong performance, achieving a rate of 93.44%. See Figure 17 and Figure 18, along with Table 4.

Upon examining the results across all metrics, it is evident that the InceptionResNetV2 and Inception UNet models achieved the highest performance in the study. In contrast, the MobileNetV2 and SegNet models were observed to produce the lowest results among their counterparts.

## 5. Discussion

As shown in Table 3 and Figure 14, Figure 15 and Figure 16, the InceptionResNetV2-based model outperformed the others across most metrics, emerging as the best overall. It not only achieved the highest accuracy but also excelled in precision, recall, and F1-Score, indicating a well-balanced performance. The accomplishment of the model can be credited to its implementation of residual mapping, which helps alleviate challenges associated with being a “very deep model” and the integration of parallel convolutional layers at the end of each inception block. This combination of techniques in InceptionResNetV2 led to the best results in this study. The only exceptions were its performance in the ROC AUC metric, where it ranked last, and its average loss, which was not the lowest.

InceptionResNetV2, despite its strong overall performance, ranks slightly lower in ROC AUC because this metric evaluates performance across all thresholds. While the model excels at the chosen threshold (0.5), its ability to maintain performance consistently across different thresholds is slightly less robust than other models. However, the difference is small and does not detract from its overall effectiveness.

However, when comparing the Inception and ResNet models, it is evident that the ResNet approach performed significantly better. ResNet152V2 consistently ranked second across all metrics and was very close to the first-placed model, with only small margins in some metrics. This highlights that residual mapping was the most effective technique among the standalone approaches in this study. Furthermore, while the inception approach was not as strong as residual mapping, it still performed exceptionally well when combined rather than used alone.

On the other hand, MobileNetV2 consistently ranked last or near last across all metrics, with significant gaps in critical metrics such as accuracy and loss. This suggests that MobileNetV2 prioritizes time performance over accuracy, which aligns with its design and purpose as a “mobile” model.

In the segmentation of CT lung images, the task focuses on classifying individual pixels rather than whole images, leading to a significant imbalance between the two types of pixels: healthy and tumor. Specifically, the tumor area in an image is typically very small compared to the entire image area, often constituting less than 10 percent of the total. This results in a substantial disparity between the number of samples from each class. Consequently, the accuracy metric can appear deceptively high, while the cross-entropy loss remains low. To properly assess the results of such segmentation tasks, precision, recall, and F1-Score are more crucial than accuracy and loss. Additionally, a metric that is typically less emphasized in classification tasks—the Jaccard index, also referred to as intersection over union (IoU)—becomes the most significant indicator of a segmentation model’s success.

As shown in Table 4 and Figure 17 and Figure 18, although the accuracy and loss results are promising, with values that are either very close or equal across models, they can be deceptive. However, when evaluating other metrics, UNet emerges as the top performer in all categories except recall. InceptionUNetV2 takes the lead in recall and comes a close second in the other metrics. The high Jaccard index values for UNet and InceptionUNetV2 indicate their strong ability to accurately classify tumor areas by distinguishing pixels. In contrast, SegNet trails behind both models, with noticeable differences across all metrics.

A high precision rate suggests that the model is less likely to incorrectly classify healthy areas as tumor regions. A high recall suggests that the model is unlikely to exclude actual tumor regions from the predicted tumor area. Given these considerations, UNet excels at accurately excluding healthy areas from the predicted tumor regions with a well-balanced evaluation, attributed to high F1-Score, while closely following InceptionUNetV2 in consistently including actual tumor areas in the predictions. As the simplest approach, UNet delivers the best overall results, with the Inception-enhanced version of UNet being a close second.

The superiority of UNet over both InceptionUNetV2 and SegNet is likely due to the simplicity of the dataset and the models’ capacities in relation to the task. UNet’s straightforward architecture effectively captures the essential patterns in the CT lung images, making it well-suited for this specific application. In contrast, the more complex models—InceptionUNetV2 and SegNet—do not demonstrate significant advantages in this context, as their additional features are not fully leveraged by the dataset. This suggests that for simpler tasks, a less complex model like UNet can yield better performance, highlighting the importance of aligning model complexity with the characteristics of the dataset.

In summary, this study highlights the critical importance of selecting appropriate models and evaluation metrics when dealing with the dual challenges of classification and segmentation in lung cancer detection. Building on the foundations laid by previous works, which have predominantly focused on either classification or segmentation independently, our approach demonstrates the value of integrating both methods. While InceptionResNetV2 proved to be the most effective in classification tasks, the UNet model excelled in segmentation, particularly in accurately identifying tumor regions. These findings underscore the need for a combined approach that leverages the strengths of different models for both classification and segmentation tasks. Future research should continue to explore hybrid models that build upon the strengths of earlier studies, integrating the robustness of classification algorithms with the precision of segmentation techniques, ultimately improving early detection and treatment planning for lung cancer.

## 6. Conclusions

Today, lung cancer remains one of the most widespread and lethal forms of cancer globally. Despite progress in treatment methods, lung cancer is difficult to treat due to its often late diagnosis. Therefore, early detection is crucial to prevent the disease from spreading. The target of this study is to recognise lung cancer in its early stages. While deep learning methods have proven highly accurate in detecting diseases from images, the InceptionResNetV2 model has demonstrated particularly impressive results, with an accuracy rate of 98.57%. Following such a strong detection, the UNet model delivers excellent precision in outlining the affected area, achieving a rate of 95.86%. Despite the fact that the research focuses on classifying healthy and diseased lungs and segmenting tumor areas, it does not discriminate between various types of tumors, even benign and malign ones. Our future goal is to develop an extended model capable of distinguishing between and segmenting these different tumor types.

## Figures and Tables

**Table 1:** Sequential layers of classification models.

Input Layer
Data Augmentation Layer
Rescaling Layer
Pre-Trained Model (one of MobileNetV2, ResNet152V2, InceptionResNetV2, Xception, VGG-19, and InceptionV3)
Pooling Layer (Global Average)
Prediction Layer (Softmax)

**Table 2:** Layers of segmentation models.

Input Layer
Encoder Layers
Bottleneck Layers
Decoder Layers
Final Convolutional Layer

**Table 3:** Metric Results of Classification.

Base Model	Accuracy	Loss	Precision	Recall	F1-Score	ROC AUC
MobileNetV2	92.32%	0.19	92.93%	92.81%	92.87%	99.43%
ResNet152V2	98.29%	0.04	98.36%	98.28%	98.32	99.71%
Xception	98.01%	**0.03**	98.02%	97.98%	98.00%	99.92%
InceptionResNetV2	**98.57%**	0.07	**98.61%**	**98.54%**	**98.58%**	99.39%
VGG-19	96.02%	0.10	96.50%	95.78%	96.14%	**99.93%**
InceptionV3	97.44%	0.09	97.54%	97.47%	97.50%	99.43%

The best result in each column (the lowest in loss and the highest in the rest) are in bold.

**Table 4:** Metric Results of Segmentation.

Models	Jaccard Index	Accuracy (%)	Loss	Precision (%)	Recall (%)	F1-Score (%)
UNet	**95.86**	**99.95**	0.001	**98.49**	97.21	**97.85**
SegNet	93.44	99.92	0.001	96.01	96.98	96.49
InceptionUNetV2	95.39	99.94	0.001	97.74	**97.43**	97.58

The best result in each column are in bold.

## Data Availability

The dataset that was used in this experiment is available at [33].

## Abbreviations

The following abbreviations are used in this manuscript:

**AI:**	Artificial intelligence
**CNN:**	Convolutional neural networks
**CT:**	Computed tomography
**ROC AUC:**	Area under the curve of receiver operating characteristic
